# A high-resolution cucumber cytogenetic map integrated with the genome assembly

**DOI:** 10.1186/1471-2164-14-461

**Published:** 2013-07-09

**Authors:** Jianying Sun, Zhonghua Zhang, Xu Zong, Sanwen Huang, Zongyun Li, Yonghua Han

**Affiliations:** 1 Institute of Integrative Plant Biology, School of Life Science, Jiangsu Normal University, Xuzhou 221116, China; 2 Key Laboratory of Horticultural Crops Genetic Improvement of Ministry of Agriculture and Institute of Vegetables and Flowers, Chinese Academy of Agricultural Sciences, Beijing 100081, China; 3 The Key Laboratory of Biotechnology for Medicinal Plants of Jiangsu Province, Jiangsu Normal University, Xuzhou 221116, China

## Abstract

**Background:**

High-resolution cytogenetic map can provide not only important biological information on genome organization but also solid foundation for genetic and genomic research. The progress in the molecular and cytogenetic studies has created the basis for developing the cytogenetic map in cucumber (*Cucumis sativus* L.).

**Results:**

Here, the cytogenetic maps of four cucumber chromosomes (chromosomes 1, 3–5) were constructed by fluorescence *in situ* hybridization (FISH) analysis on cucumber pachytene chromosomes. Together with our previously constructed cytogenetic maps of three cucumber chromosomes (chromosomes 2, 6–7), cucumber has a complete cytogenetic map with 76 anchoring points between the genetic, the cytogenetic and the draft genome assembly maps. To compare our pachytene FISH map directly to the genetic linkage and draft genome assembly maps, we used a standardized map unit—relative map position (RMP) to produce the comparative map alignments. The alignments allowed a global view of the relationship of genetic and physical distances along each cucumber chromosome, and accuracy and coverage of the draft genome assembly map.

**Conclusions:**

We demonstrated a good correlation between positions of the markers in the linkage and physical maps, and essentially complete coverage of chromosome arms by the draft genome assembly. Our study not only provides essential information for the improvement of sequence assembly but also offers molecular tools for cucumber genomics research, comparative genomics and evolutionary study.

## Background

Cucumber (*Cucumis sativus* L*.*, 2n = 2x = 14), which belongs to the family Cucurbitaceae, is an economically important crop as well as a model system to study biologically relevant characters such as sex determination [1] and plant vascular biology [2]. For these reasons, sequencing of the cucumber genome and the development of functional genomic tools are of great importance. Since Huang et al. [3] first reported the draft genome sequence of the ‘Chinese long’ inbred line ‘9930’ (database is hosted at http://cucumber.genomics.org.cn/page/cucumber/index.jsp), other two cucumber lines (the North American pickling type inbred line ‘Gy14’ and the North-European Borszczagowski cultivar ‘B10’ ) were also sequenced [4,5]. Based on the genetic maps, draft genome assemblies were developed in the three lines [3,5,6]. In inbred line 9930, 193.3 Mbp of the assembled sequences were anchored onto the chromosomes to generate the draft genome assembly based on the genetic map developed by Ren et al. [7]. However, the quality of the 9930 genome assembly has not been validated. A high-quality genetic map is necessary to the draft genome assembly. The mapping population used in Ren et al. [7] was derived from the inter-subspecific cross between the cultivated cultivar Gy14 and the wild accession PI 183967. Due to marker clustering resulted by structure rearrangements between Gy14 and PI 183967, mapping distance (cM) on chromosomes 4, 5 and 7 was dramatically less than that detected on other four chromosomes. Moreover, although genetic linkage map is usually good indicator of the marker order, the exact physical positions of the genetic loci and genomic sequences on the chromosomes are unknown. This is because crossovers are not equally distributed over chromosome arms, and as a result loci that are physically far apart on chromosomes can be tightly linked on linkage maps and vice versa. Such discrepancies are impediments to applying linkage maps to guide genome sequence assembly.

The molecular cytogenetic map constructed by localizing marker-tagged clones directly on pachytene chromosomes by FISH method provides directly visible physical positions of the associated molecular markers along a given chromosome [8]. Such map combines chromosome structure with recombination rate and physical distance, thus providing integrated biological information on genome organization. To date, high-resolution cytogenetic maps are available for individual chromosomes in maize [9,10], rice [11,12], *Brassica*[13,14], tomato [15-17], soybean [18], cotton [19] and papaya [20], and for the whole genome chromosomes in *Sorghum*[21], potato [22-24], common bean [25,26] and cucumber inbred line ‘Gy14’ [6]. The molecular cytogenetic maps of three cucumber chromosomes in inbred line 9930 were constructed, correlating physical and genetic distances, characterizing the distribution of the heterochromatic regions in the chromosome complement, as well as conducting comparative mapping to melon chromosomes in our previous studies [27,28]. No such cytogenetic maps are currently available, however, for other four cucumber chromosomes.

The different kinds of maps differ greatly in method of production, units and the ways they are viewed. Integrating different map types with shared markers will provide a comprehensive view of genome structure. The loci positions for the pachytene cytogenetic maps are usually charted in fraction length (FL: the percentage of the distance from the FISH site to the end of the short arm relative to the total length of the chromosome). To intuitively display the relationship between the genetic and physical distances, the positions of FISH mapped loci were transformed into the product of fraction length and the total length (cM) of corresponding linkage group in some studies [12,22,27,28]. Figueroa and Bass [10] used relative map position (RMP) unit, which was the percentage distance of a locus from the centromere along a given chromosome arm, to compare the different maps of maize directly. This provides a new means for intuitively comparing the cytogenetic, linkage, and physical maps of maize.

In the present work, we constructed the molecular cytogenetic maps of the remaining four cucumber chromosomes (chromosomes 1, 3–5) by FISH analysis using 8–14 fosmid clones per chromosome. These fosmid clones distributed at regular intervals across the chromosome-level cucumber draft genome assembly maps and some clones carried major genetic markers. Together with our previously published data [27,28], cucumber inbred line 9930 has a complete molecular cytogenetic map with 76 FISH mapped loci. Referring to the method used by Figueroa and Bass [10], we also used similar relative map position (RMP) units, which was the percentage distance of a locus from the end of the short arm along a given chromosome, for direct comparative analysis between the cytogenetic, the genetic linkage, and draft genome assembly maps of cucumber.

## Results

### The construction of molecular cytogenetic maps of cucumber chromosomes 1, 3–5

**Figure 1:**
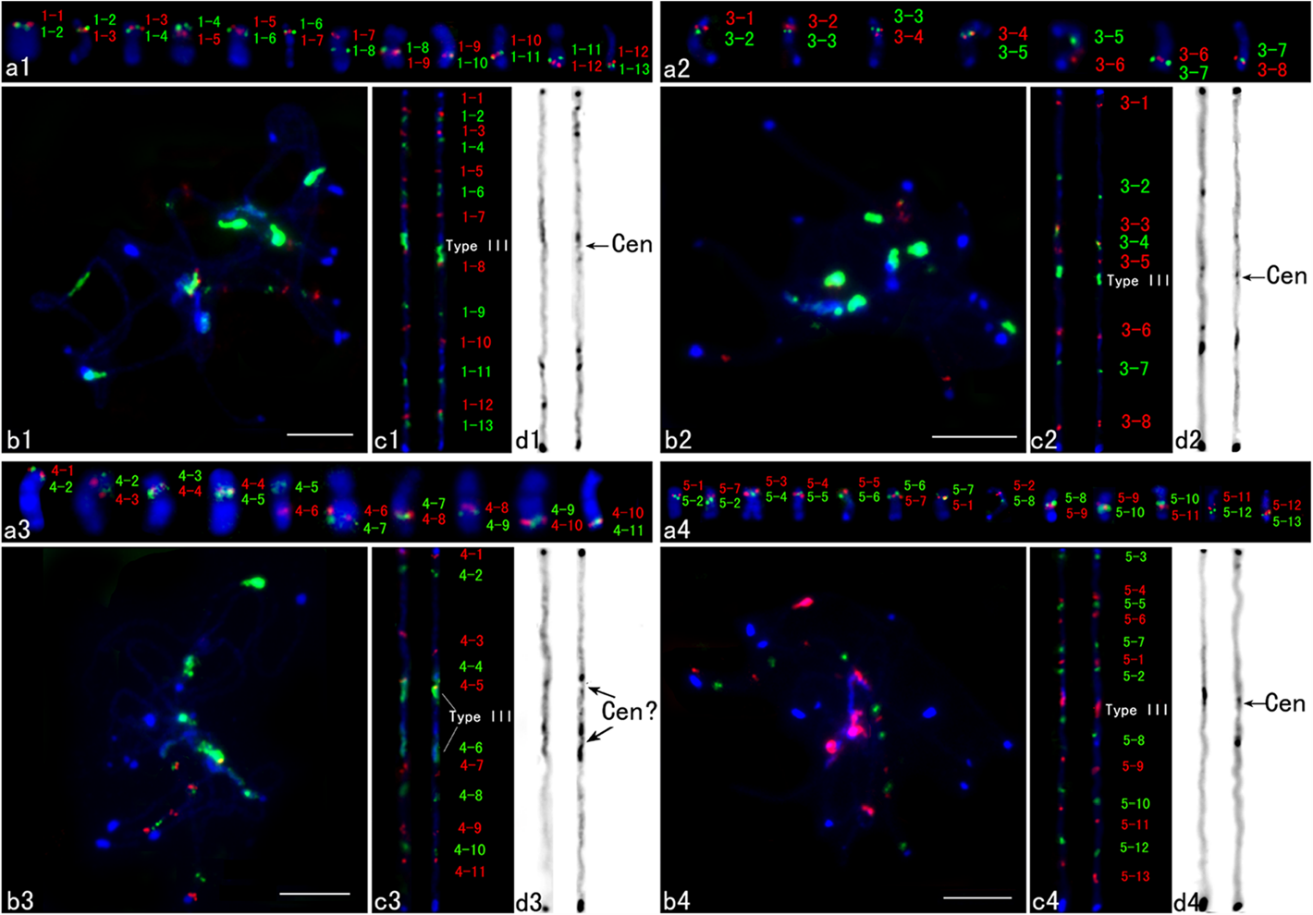
**FISH of fosmids on cucumber somatic and pachytene chromosomes 1, 3–5. a1**-**a4** FISH of fosmids on somatic chromosomes. Adjacent probes were labeled with different fluorochromes and hybridized together to test the order and position. **b1**-**b4** Cucumber chromosomes at the pachytene stage were probed by a set of fosmid clones together with the Type III satellite repeat. **c1**-**c4** Two straightened cucumber pachytene chromosomes. The left chromosome was straightened from the image shown in Figures **b1**-**b4**. **d1**-**d4** The chromosomes in Figures **c1**-**c4** were converted into black-white image. *Bars*, 5 μm.

To construct the molecular cytogenetic maps of the remaining four cucumber chromosomes, we selected a set of fosmid clones distributed at regular intervals across the chromosome-level cucumber draft genome assembly maps. We first determined the physical order of adjacent fosmid clones based on their positions in draft genome assembly map by dual-color FISH on somatic metaphase chromosomes (Figures 1a1-a4). On the basis of these results, multi-fosmid FISH probe cocktails were developed and hybridized to the pachytene chromosomes together with the cucumber centromere-specific DNA probe Type III (Figures 1b1-b4). The cocktails produced alternate red/green signals of all clones and marked centromeres on each chromosome. Although the pachytene cucumber chromosomes are usually tangled with each other, which makes it difficult to trace individual chromosomes. We were able to strip the signals derived from multi-fosmid clone cocktails and confirm chromosomes. Two computationally straightened chromosomes from two independent cells were shown (Figures 1c1-c4). The left chromosome was straightened from the image shown in Figures 1b1-b4.

A total of 16 fosmid clones distributed at an average distance of 1.68 Mbp along the cumulative physical length (29149675 bp) of the chromosome 1 were selected for FISH mapping. Among these 16 clones, we confirmed that 13 clones (1–1 to 1–13) showed unique hybridization signals on mitotic metaphase chromosomes by FISH (see Additional file 1, Figure 1a1). The remaining 3 clones which showed repetitive FISH signals were not used (data not shown). Thirteen fosmid probes together with the centromere-specific DNA probe Type III were chosen to make a multicolor FISH cocktail mix. The relative position of all probes can be clearly distinguished on spreads of pachytene bivalents (Figure 1b1). Relative to a centromere repeat probe Type III, the short arm of chromosome 1 was identified by the hybridization of 7 fosmid clones and the remaining 6 clones hybridized to the long arm of chromosome 1 (Figure 1c1).

Eleven fosmid clones distributed at an average distance of 3.66 Mbp across the chromosome 3 (39782674 bp) were selected for FISH mapping. Two clones which showed repetitive FISH signals in mitotic metaphase chromosomes were discarded (data not shown), one clone (Ch3-1) yielded strong signals on other chromosomes but not signals on chromosome 3 was also discarded (see Additional file 2 and Additional file 3a). The remaining 8 clones (3–1 to 3–8) with unique hybridization signals were used for FISH mapping on the pachytene chromosomes together with the Type III probe (see Additional file 1, Figure 1a2-b2). The centromere was located between fosmids 3–5 and 3–6 (Figure 1c2).

Thirteen fosmids distributed at an average distance of 1.95 Mbp on chromosome 4 (physical length: 23425844 bp) were selected for FISH mapping. Two clones which showed repetitive FISH signals in mitotic metaphase chromosomes were discarded (data not shown). The remaining 11 (4–1 to 4–11) with unique hybridization signals were chosen to make a multicolor FISH cocktail mix together with the probe Type III (see Additional file 1, Figure 1a3-b3). Interestingly, Type III sequence hybridized to two regions located between fosmids 4–5 and 4–6 on chromosome 4 and we cannot identify its functional centromere region at present (Figure 1c3).

A total of 21 fosmid clones distributed at an average distance of 1.52 Mbp on chromosome 5 (physical length: 28023477 bp) were selected. 6 clones which showed repetitive FISH signals in mitotic metaphase chromosomes were discarded (data not shown), one clone (Ch5-1) showed strong FISH signals on other chromosome pair but not signals on chromosome 5 was discarded (Additional file 2, Additional file 3b). One clone (Ch5-2) which was assembled on long arm in genomic sequence map but showed FISH signals on short arm of chromosome 5 was also discarded (see Additional file 2, Additional file 3c). The remaining 13 clones (5–1 to 5–13) with unique hybridization signals were used for FISH mapping (Additional file 1). The order of individual fosmids along chromosome 5 was generally concordant with the order on the genomic sequence map, except that fosmids 5–1 and 5–2 showed different positions (Figure 1a4-c4). The centromere was located between fosmids 5–2 and 5–8 (Figure 1c4).

**Figure 2:**
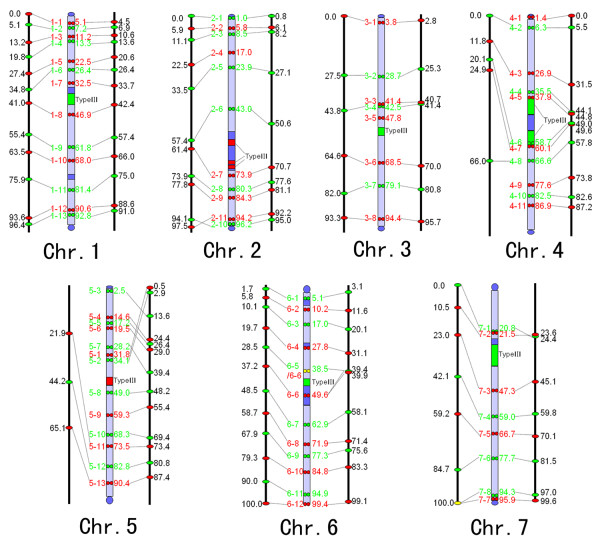
**Integrated genetic (left)/cytogenetic (middle)/genome assembly (right) maps of cucumber inbred line 9930.** Ideograms of cytogenetic maps show physical locations of heterochromatin, fosmid clones and the centromere repeat Type III. Clones are indicated in red and green, and chromosomes in light blue. Dark blue blocks represent approximate locations of constitutive heterochromatin. Numbers in each map indicate the RMPs of FISH-mapped loci.

The DAPI-stained pachytene chromosomes in Figures 1c1-c4 were converted into a black-white image to show heterochromatic distribution. Figures 1d1-d4 displayed converted images of these chromosomes, which were straightened and stretched to equal length and slightly sharpened for better heterochromatin differentiation. The dark blocks represented the dense brightly fluorescing heterochromatin regions, whereas the lighter regions were euchromatic regions showed the fainter DAPI signal intensity. Based on the DAPI staining, four cucumber chromosomes showed different heterochromatin and euchromatin distribution patterns, although similar heterochromatin domains were observed at both ends of each chromosome (Figures 1d1-d4). To intuitively display the distributions and positions of heterochromatin and fosmid clones on four cucumber chromosomes, ideograms were constructed based on measure on the same 5 pachytene chromosomes without morphology distortion showing all hybridization spots. The relative map position of individual fosmid and the length of heterochromatin region along pachytene chromosome were calculated and the results were listed in Additional file 1, Additional file 4 and Figure 2.

### Integration and analysis of clone positions across three cucumber maps

To compare our pachytene FISH map directly to the genetic linkage map and draft genome assembly map, we used a standardized map-unit system in which the percentage distance of each locus along the chromosome in each map was used, denoted as RMP units. The RMP values of FISH-mapped loci on all cucumber chromosomes based on this study and our previous works [27,28] were summarized in Additional file 1. Figure 2 showed composite alignments of three maps. The alignments allowed a global view of the relations between the genetic positions of the corresponding anchoring SSR markers, chromosomal positions and physical positions in draft genome map of the fosmid clones.

In comparing the pachytene FISH map to the genetic linkage map [7], we found that the linear order of markers in the linkage groups was in complete agreement with the order of the corresponding fosmid clones along the chromosomes except for 2–10 and 2–11 (Figure 2). Although the majority of loci showed good RMP agreement, the RMPs showed considerable variation (differing by more than 10 RMP units) in some loci on chromosomes 2 (2–6, 2–7), 4, 5, 6 (6–6 to 6–8) and 7 (7–1 to 7–4). For example, 5–11 showed a maximum difference of 51.6 RMP units. When marker intervals were compared between two maps, some discrepancies were detected between genetic distance and physical distance. Differences were particularly obvious (more than 10 RMP units) for marker intervals spanning the centromere regions of chromosomes 1, 2, 4 and 7. The markers flanking the centromeres were separated by short genetic distance but long physical distance. The reduction of recombination around the centromere is a common feature and the region of recombination suppression correlates directly with sizes of centromeric heterochromatic regions. Obvious region of recombination suppression was not detected on chromosomes 3, 5–6 having small sizes of centromeric heterochromatin regions. In non-centromeric regions, recombination was basically evenly distributed along the physical length of chromosome.

We next compared the distributions of fosmid clones on the 9930 draft genome assembly map and our cytogenetic map (Figure 2). The RMPs were remarkably similar, showing comparable distributions along the given chromosomes (Figure 2). The RMP differences were less than 10 RMP units except for the loci on the short arm of chromosome 5. Loci mapped closer to the centromeres exhibited the greater RMP difference between the maps. The linear orders of the loci along a given chromosome were congruent between two maps except for 5–1 and 5–2, 7–1 and 7–2. One criterion to judge the quality of a draft genome assembly map is its physical coverage of the corresponding chromosome. The cucumber chromosomal ends and centromeres were occupied by several tandem repeat sequences **(**Type I/II, Type III and Type IV) [29]. Each of these repeat classes exists as large tandem arrays which pose significant technical challenges in assembly accuracy. Thus, these chromosomal regions were all left as “gaps” in the draft genome sequence map. We founded that the FISH signals of fosmid clones selected from the distal ends of each pseudo-chromosome in draft genome assembly map were indeed physically located at the telomeric ends of each chromosome. Except for the interval between 6–5 and 6–6, RMP intervals were similar to the clones flanking the centromeres between two maps when the lengths of Type III regions weren’t considered. No large gaps were founded in pericentromeric regions in draft genome assembly map. The physical positions of the anchored fosmids along all chromosomes correlate well with their positions in the genome sequence map. These results showed that draft genome assembly map covered almost the entire physical length of cucumber chromosomes.

## Discussion

While a total of 72.2-fold genome coverage was generated for the cucumber genome. However, the total length of the assembled cucumber genome was only 243.5 Mb, about 30% smaller than the 367 Mbp cucumber genome. Consistent with this proportion, several types of satellite sequences accounted for about 20-30% of the total nuclear DNA in cucumber based on the proportion of rDNA (3.3% of the genome), Type III (4.04%), Type I/II and Type IV (15.92%) on mitotic chromosomes [3,29]. These repeat classes exists as large tandem arrays, likely in the form of higher-order repeat units of slight variants of the main consensus repeat which pose significant technical challenges in assembly accuracy [30,31]. The tandem arrangement could be left aside during a genome assembly endeavor. Even the most rigorous clone-by-clone sequencing approach has not yielded data on the complete DNA sequences of a centromere from any higher plant or animal species, which have often abundant satellite DNA extending over several hundreds of thousands or millions of base pairs. Therefore, the majority of the remaining 30% of unassembled genome are likely to satellite sequences.

Mis-assemblies are common when draft genome sequences have been generated by de novo assembly of sequences obtained with NGS technologies [32,33]. Since the assembly of inbred line 9930 was done using the SOAPdenovo software from mostly Illumina reads (68.36 coverage) together with “Sanger” generated genomic library end sequences (only 3.96 coverage), mis-assembled scaffolds may exist in the draft genome. For example, Yang et al. [6] identified five mis-assembled scaffolds from the 9930 draft genome and their positions were verified by FISH. However, so far, it did not incorporate complete cytogenetic mapping data to assess contiguity, collinearity and coverage of the 9930 draft genome. In this study, we allocated some fosmid clones from each chromosme to pachytene spreads by FISH method, these fosmid clones distributed at regular intervals across the chromosome-level cucumber draft genome assembly maps and some clones carried major genetic markers. Although most clones yielded signals on the corresponding chromosomes, we identified seven misassembled clones which three clones yielded signals on other chromosome(s) or chromosome arm (Ch3-1, Ch5-1, Ch5-2) and four clones (5–1 and 5–2, 7–1 and 7–2) showed wrong positions on same chromosome arm. We examined the distribution of misassembled clones in cucumber inbred line Gy14 by searching the Gy14 genome assembly using the sequence of each misassembled fosmid clone. We founded that the location and order of 5–1 and 5–2 were same in Gy14 genome and the 9930 cytogenetic map of chromosome 5. Clearly, the 5–1 and 5–2 were misassembled in the 9930 draft genome assembly map. However, the locations of other clones (Ch3-1, Ch5-1, Ch5-2, 7–1 and 7–2) were same in Gy14 and 9930 genome assembly maps. This may be because these clones were misassembled in 9930 and Gy14 genome assembly maps. Of course, it cannot be excluded that we used the wrong clones. More mis-assemblies may exist in the 9930 draft genome map. But nonetheless, our present results showed that the 9930 draft genome assembly map provided excellent coverage of the corresponding chromosomes and no large gaps were founded in draft genome.

The present developed molecular cytogenetic map integrated with genetic linkage map and physical map not only verifies the quality of 9930 draft genome assembly and provides essential information for the improvement of cucumber genome assembly but also offers molecular tools for cucumber genomics research. The majority FISH-mapped fosmid clones carried major genetic markers thus our analysis revealed the genetic and physical relationships in specific chromosome regions. This can provide crucial physical information to positional cloning projects that might otherwise be fruitlessly aimed at a target gene on the basis of markers that are very tightly linked but physically distant. In addition, the integrated molecular cytogenetic map also forms a solid foundation for future FISH-based comparative genomics and evolutionary studies. The reference set of fosmid clones can serve as a universal set of cytogenetic markers to study synteny and chromosomal rearrangements between cucumber and other cucurbit genomes like our previous work [27].

## Conclusions

The cytogenetic map incorporating genetic, cytological and physical data can contribute significantly to the improvement of sequence assembly by confirming the physical positions of markers on the linkage groups, identifying mis-assembled clones and evaluating the size of the putative remaining gaps. Such map also offers molecular tools for cucumber genomics research, comparative genomics and evolutionary study. In the present work, we constructed the molecular cytogenetic maps of four cucumber chromosomes. Together with our previous results, cucumber has a complete molecular cytogenetic map. Furthermore, we used a standardized map unit—relative map position (RMP) to produce the comparative map alignments. The alignments showed that draft genome assembly map provided excellent coverage of the corresponding chromosomes. The reference set of fosmid clones can serve as a universal set of cytogenetic markers for comparative genomics study between cucumber and its close relatives.

## Methods

### Plant materials and chromosome preparation

*C. sativus* ‘Chinese long’ inbred line 9930 was used for cytological studies. Root tips were harvested from germinated seeds, pretreated in 0.002 M 8-hydroxyquinoline at room temperature for 2 h to accumulate metaphase cells, and fixed in methanol:glacial acetic acid (3:1). Root tips were macerated in 2% cellulase Onozuka R-10 (Yakult Pharmaceutical, Tokyo) and 1% pectolyase Y-23 (ICN) at 37°C for 2 h and squashes were made in the same fixative. Young panicles were harvested and fixed in 3:1 (100% ethanol:glacial acetic acid) Carnoy’s solution. The procedure for meiotic chromosome preparation was largely the same as that used for preparing mitotic chromosomes from root tips with the following modification: anthers were digested in the enzyme mixture for 4.5 h at 37°C. The digested anthers were macerated on glass slides in 50% acetic acid solution with fine-pointed forceps and then “flame-dried” over an alcohol flame.

### Fluorescence *in situ* hybridization (FISH) and cytological measurements

All fosmid clones were provided by the Institute of Vegetables and Flowers, Chinese Academy of Agricultural Sciences. The fosmid clones screened from the 9930 fosmid library distributed at regular intervals across the chromosome-level cucumber draft genome assembly maps and some clones carried major genetic markers. Fosmid DNA was isolated using QIAGEN plasmid midi kit and further purified by Plant DNeasy spin columns (QIAGEN). The centromere-specific DNA probe Type III repeat [29] was used. FISH was performed according to Jiang et al. [34]. DNA probes were labeled with digoxigenin-dUTP or biotin-dUTP via nick translation and detected with antidigoxigenin antibody coupled with Rhodamine (Roche) or avidin-conjugated with FITC (Vector Laboratories), respectively. Chromosomes were counterstained by 4,6-diamidino-2-phenylindole (DAPI) in a VectaShield antifade solution (Vector Laboratories). Images were captured digitally using a CCD camera (QIMAGING, RETIGA-SRV, FAST 1394) attached to an Olympus BX63 epifluorescence microscope. Gray-scale images were captured for each color channel and then merged. Chromosome straightening was performed using the ‘straighten-curved-objects’ plug-in of Image J [35], and measurements were made on the digital images of the FISH signals and chromosomes using Image-Pro Plus 7.0C software (Media Cybernetics) and final image adjustments were done with Adobe Photoshop (Adobe Systems).

### Comparative mapping using standardized map units

We used relative map position (RMP) units for direct comparative analysis between the cytogenetic, the genetic linkage, and draft genome assembly maps of cucumber. The RMP values for the pachytene cytogenetic map were the percentage of the distance (in μm) from the FISH site to the end of the short arm relative to the total length of the chromosome (in μm). In order to establish the position of each clone along the chromosomes, hybridization signals on the same 5 pachytene chromosomes without morphology distortion showing all hybridization spots were measured. The RMP values for the SSR linkage map [7] were the percentage from the genetic location (cM) of each locus along the total length (cM) of the corresponding linkage group. The RMP values for the 9930 draft genome assembly map were calculated from the genomic location (bp) of each locus along the cumulative physical length of chromosomes 1 to 7 (http://cucumber.genomics.org.cn/). These RMP values were used to produce the comparative map alignments.

## Competing interests

The authors declare that they have no competing interests.

## Authors’ contributions

YHH designed the study, performed the analysis and wrote the paper. SWH and ZYL participated in the design of the study, analyzed the data and critically revised the manuscript. JYS, ZHZ and XZ carried out the experiments and acquisition of original data. JYS analyzed the data and participated in writing. All authors read and approved the final manuscript.

## Supplementary Material

Additional file 1 FISH-mapped fosmid clones and their corresponding RMPs in genetic, cytogenetic, and draft genome assembly maps. Click here for file

Additional file 2 Positions of three identified misassembled clones. Click here for file

Additional file 3 **FISH results of three identified misassembled clones. a** The signals of Ch3-1 (red) and 3–2 (green) weren’t in the same chromosome pair. **b** The signals of Ch5-1 (red) and 5–10 (green) weren’t in the same chromosome pair. **c** Ch5-2 (red) was not in the long arm with 5–11 signals but in the short arm of chromosome 5. Click here for file

Additional file 4 Heterochromatin distribution on cucumber pachytene chromosomes 1, 3–5. Click here for file
