# Supplementary material for: A high-resolution cucumber cytogenetic map integrated with the genome assembly
Source: BMC Genomics. 2013 Jul 9;14:461. doi: 10.1186/1471-2164-14-461 (PMC3710503; doi:10.1186/1471-2164-14-461)
Supplement: Additional file 1 — FISH-mapped fosmid clones and their corresponding RMPs in genetic, cytogenetic, and draft genome assembly maps. [file 1471-2164-14-461-S1.doc]

| Code | **Marker** | **Position (cM)** | **Genome Position (bp)*^a^*** | Fosmid clone | **Relative map positions (%)** | | |
| --- | --- | --- | --- | --- | --- | --- | --- |
|  |  |  |  |  | **Genetic** | **Cytogenetic** | **Genome** |
| 1-1 | SSR13109 | 0.0 | 1297220- 1332090 | gcfbd0_0248_H03.ab1 | 0.0 | 5.1±0.5 | 4.5 |
| 1-2 | SSR23757 | 4.9 | 1966203- 2006434 | gcfbd0_0464_C11.ab1 | 5.1 | 7.2±0.2 | 6.9 |
| 1-3 | SSR05793 | 12.7 | 3077660- 3113010 | gcfbe0_0480_D12.ab1 | 13.2 | 11.2±0.2 | 10.6 |
| 1-4 | SSR12157 | 19.0 | 3964701- 3997356 | gcfaa0_0041_G09.ab1 | 19.8 | 13.3±0.3 | 13.6 |
| 1-5 | SSR05501 | 26.326 | 6011215- 6046796 | gcfbd0_0358_H04.ab1 | 27.4 | 22.5±0.4 | 20.6 |
| 1-6 | SSR12070 | 33.502 | 7698959- 7732033 | gcfbe0_0544_G05.ab1 | 34.8 | 26.4±0.4 | 26.4 |
| 1-7 | — | — | 9821047- 9821197 | gcfbe0_0205_D01.ab1 | — | 32.5±0.9 | 33.7 |
| 1-8 | SSR07405 | 39.4 | 12352419- 12390384 | rgcfbe0_0096_H01.ab1 | 41.0 | 46.9±1.5 | 42.4 |
| 1-9 | SSR17196 | 53.3 | 16721315- 16751338 | gcfbd0_0990_G07.ab1 | 55.4 | 61.8±1.4 | 57.4 |
| 1-10 | SSR15575 | 61.053 | 19243863- 19283488 | gcfbe0_0148_C12.ab1 | 63.5 | 68.0±1.6 | 66.0 |
| 1-11 | SSR14445 | 73.029 | 21848915- 21879630 | gcfba0_0007_C04.ab1 | 75.9 | 81.4±0.2 | 75.0 |
| 1-12 | SSR00262 | 90.05 | 25819260- 25853168 | gcfbe0_0309_G05.ab1 | 93.6 | 90.6±0.1 | 88.6 |
| 1-13 | SSR00129 | — | 26523245- 26556632 | gcfbd0_1002_C05.ab1 | 96.4 | 92.8±0.1 | 91.0 |
| 2-1 | SSR00184 | 0 | 142789-184478 | gcfbe0_0022_B06.ab1 | 0.0 | 1.0±0.1 | 0.8 |
| 2-2 | SSR11952 | 5.9 | 1374855-1409777 | gcfbd0_1078_H03.ab1 | 5.9 | 5.8±0.6 | 6.1 |
| 2-3 | SSR21090 | 11.1 | 1858360-1887141 | gcfbd0_0606_B07.ab1 | 11.1 | 8.5±0.9 | 8.2 |
| 2-4 | SSR13504 | 22.5 | — | gcsxc0_157514 | 22.5 | 17.0±0.6 | — |
| 2-5 | SSR22083 | 33.6 | 6251440-6285298 | gcfbd0_0656_E02.ab1 | 33.5 | 23.9±0.2 | 27.1 |
| 2-6 | SSR03758 | 57.5 | 11687924-11722220 | rgcfbe0_0464_B06.ab1 | 57.4 | 43.0±0.4 | 50.6 |
| 2-7 | SSR23732 | 61.5 | 16344196-16377413 | rgcfbd0_0252_E04.ab1 | 61.4 | 73.9±0.7 | 70.7 |
| 2-8 | SSR20045 | 74 | 17928507-17965119 | gcfbd0_1142_B06.ab1 | 73.9 | 80.3±0.6 | 77.6 |
| 2-9 | SSR06678 | 78 | 18762808-18796796 | rgcfbd0_0512_G07.ab1 | 77.8 | 84.3±0.4 | 81.1 |
| 2-10 | SSR30665 | 94.3 | 21968920-22001624 | gcfbd0_0304_A09.ab1 | 94.1 | 96.2±0.3 | 95.0 |
| 2-11 | SSR13783 | 97.7 | 21344140-21370552 | gcfbd0_0605_C06.ab1 | 97.5 | 94.2±0.1 | 92.2 |
| 3-1 | SSR03049 | 0 | 1121698- 1155015 | rgcfbe0_0472_B12.ab1 | 0.0 | 3.8±0.1 | 2.8 |
| 3-2 | SSR22514 | 31 | 10059164- 10101377 | gcfbe0_0207_F03.ab1 | 27.5 | 28.7±1.1 | 25.3 |
| 3-3 | SSR02244 | — | 16190322-21441020 | gcfbd0_0008_A02.ab1 | — | 41.4±0.8 | 40.7 |
| 3-4 | CSWGATT01B | 49.4 | 16484183- 16510660 | gcfbd0_0554_G09.ab1 | 43.8 | 42.5±0.7 | 41.4 |
| 3-5 | SSR14526 | — | — | gcfbd0_0392_E05.ab1 | — | 47.8±0.6 | — |
| 3-6 | SSR21008 | 72.8 | 27831362- 27875574 | gcfbd0_0609_D07.ab1 | 64.6 | 68.5±0.2 | 70.0 |
| 3-7 | SSR21454 | 92.4 | 32147183- 32188786 | gcfbe0_0104_H02.ab1 | 82.0 | 79.1±0.6 | 80.8 |
| 3-8 | SSR23517 | 105.1 | 38050645- 38088342 | gcfbe0_0001_D11.ab1 | 93.3 | 94.4±0.3 | 95.7 |
| 4-1 | — | — | 351- 43490 | gcfbe0_0315F11 | — | 1.4±0.5 | 0.0 |
| 4-2 | SSR03598 | 0 | 1301733- 1335906 | gcfbd0_0280_E09.ab1 | 0.0 | 6.3±0.2 | 5.5 |
| 4-3 | SSR01601 | 4.4 | 7374241- 7410345 | gcfbe0_0162_A08.ab1 | 11.8 | 26.9±1.7 | 31.5 |
| 4-4 | — | — | 10327544- 10359112 | gcfbd0_0108G02 | — | 35.5±1.2 | 44.1 |
| 4-5 | — | — | 10490315- 10524387 | gcfbd0_0476H06.ab1 | — | 37.9±1.3 | 44.8 |
| 4-6 | SSR23826 | 7.5 | 11480614- 11510697 | gcfbe0_0243_E12.ab1 | 20.1 | 58.7±0.9 | 49.0 |
| 4-7 | SSR14617 | 9.3 | 11633420- 11670457 | gcfba0_0090_G08.ab1 | 24.9 | 60.1±0.2 | 49.6 |
| 4-8 | SSR05125 | 24.6 | 13550645- 13580918 | gcfbe0_0082_D04.ab1 | 66.0 | 66.6±0.1 | 57.8 |
| 4-9 | — | — | 17279631- 17313906 | gcfbd0_0245F04 | — | 77.6±0.7 | 73.8 |
| 4-10 | — | — | 19347769- 19383016 | gcfbe0_0098_F07.ab1 | — | 82.5±0.3 | 82.6 |
| 4-11 | — | — | 20434117- | gcfbd0_0077_A03.ab1 | — | 86.9±0.3 | 87.2 |
| 5-1 | — | — | 138737- 170257 | gcfbd0_0427D01 | — | 31.8±0.1 | 0.5 |
| 5-2 | — | — | 808885- 847462 | gcfbd0_0122G12 | — | 34.1±0.1 | 2.9 |
| 5-3 | — | — | 3813355- 3851250 | gcfbd0_0986F03 | — | 2.5±0.3 | 13.6 |
| 5-4 | — | — | 6835282- 6873999 | gcfba0_0036C03 | — | 14.6±0.1 | 24.4 |
| 5-5 | — | — | 7390011- 7427811 | gcfbd0_0696F11 | — | 17.2±0.2 | 26.4 |
| 5-6 | — | — | 8124088- 8155885 | gcfbd0_0763F06 | — | 19.5±0.2 | 29.0 |
| 5-7 | — | — | 11041510- 11076720 | gcfbe0_0314B09 | — | 28.2±0.2 | 39.4 |
| 5-8 | — | — | 13498662- 13531056 | gcfbd0_0837E12 | — | 49.0±0.1 | 48.2 |
| 5-9 | — | — | 15517174- 15552093 | gcfbd0_0041E07 | — | 59.3±0.3 | 55.4 |
| 5-10 | — | — | 19447820- 19476637 | gcfbe0_0229A07 | — | 68.3±0.2 | 69.4 |
| 5-11 | SSR11439 | 13.1 | 20584946- 20616763 | gcfbd0_0222_H10.ab1 | 21.9 | 73.5±0.3 | 73.4 |
| 5-12 | SSR21918 | 26.5 | 22654705- 22698074 | rgcfbe0_0257_D06.ab1 | 44.2 | 82.8±0.1 | 80.8 |
| 5-13 | SSR17975 | 39 | 24499019- 24533838 | gcfba0_0066_B05.ab1 | 65.1 | 90.4±0.1 | 87.4 |
| 6-1 | SSR00158 | 1.8 | 873778-909701 | gcfbe0_0620_F09.ab1 | 1.7 | 5.1±0.1 | 3.1 |
| 6-2 | SSR11219 | 6.2 | 3346943-3384961 | gcfbe0_0187_G09.ab1 | 5.8 | 10.2±0.2 | 11.6 |
| 6-3 | SSR01903 | 10.8 | 5816959-5853441 | gcfbe0_0459_C08.ab1 | 10.1 | 17.0±0.4 | 20.1 |
| 6-4 | SSR02385 | 21 | 9005744-9043443 | gcfbe0_0380_E04.ab1 | 19.7 | 27.8±0.6 | 31.1 |
| 6-5 | SSR00647 | 30.4 | 11432808-11468241 | rgcfbe0_0199_B05.ab1 | 28.5 | 38.5±2.3 | 39.4 |
| 6-6 | SSR02086 | 39.6 | 11564298-11594450 | gcfbd0_0868_F07.ab1 | 37.2 | 38.5±2.3/  49.6±3.6 | 39.9 |
| 6-7 | SSR00300 | 51.6 | 16862173- | gcfbb0_0020_B11.ab1 | 48.5 | 62.9±2.6 | 58.1 |
| 6-8 | SSR15199 | 62.5 | 20718912-20754913 | rgcfbd0_0502_G07.ab1 | 58.7 | 71.9±0.3 | 71.4 |
| 6-9 | SSR13741 | 72.3 | 21942917-21979714 | gcfbe0_0325_A08.ab1 | 67.9 | 77.3±0.1 | 75.6 |
| 6-10 | SSR02906 | 84.5 | 24183018-24219723 | rgcfbe0_0257_E12.ab1 | 79.3 | 84.8±0.3 | 83.3 |
| 6-11 | SSR22498 | 95.8 | — | gcfbd_0199_C06.ab1 | 90.0 | 94.9±0.4 | — |
| 6-12 | SSR06271 | 106.5 | 28782091-28821527 | gcfbd0_0131_G09.ab1 | 100.0 | 99.4±0.1 | 99.1 |
| 7-1 | SSR15477 | 0 | 4651252-4689593 | gcfbe0_0624_A03.ab1 | 0.0 | 20.8±1.1 | 24.4 |
| 7-2 | SSR06585 | 6.3 | 4501235-4535070 | rgcfba0_0055_H02.ab1 | 10.5 | 21.5±0.6 | 23.6 |
| 7-3 | SSR04584 | 13.8 | 8714177-8680867 | rgcfbe0_0058_F08.ab1 | 23.0 | 47.3±3.6 | 45.1 |
| 7-4 | SSR11665 | 25.3 | 11469302-11499606 | gcfbd0_0393_E02.ab1 | 42.1 | 59.0±2.8 | 59.8 |
| 7-5 | SSR22097 | 35.6 | 13449436-13483725 | gcfbd0_0214_B02.ab1 | 59.2 | 66.7±3.0 | 70.1 |
| 7-6 | SSR01898 | 50.9 | 15641671-15673977 | gcfbd0_0343_F07.ab1 | 84.7 | 77.7±2.0 | 81.5 |
| 7-7 | SSR20122 | 60.1 | 19119616-19152185 | rgcfbe0_0480_A04.ab1 | 100.0 | 95.9±0.9 | 99.6 |
| 7-8 | SSR17062 | 60.1 | 18622816-18657431 | gcfbd0_0556_D09.ab1 | 100.0 | 94.3±0.7 | 97.0 |
