# Supplementary material for: A high-resolution cucumber cytogenetic map integrated with the genome assembly
Source: BMC Genomics. 2013 Jul 9;14:461. doi: 10.1186/1471-2164-14-461 (PMC3710503; doi:10.1186/1471-2164-14-461)
Supplement: Additional file 2 — Positions of three identified misassembled clones. [file 1471-2164-14-461-S2.doc]

| Code | **Marker** | **Position (cM)** | **Genome Position (bp)*^a^*** | Fosmid clone |
| --- | --- | --- | --- | --- |
| Ch3-1 | SSR13274 | 40.7 | 12493789 - 12524545 | gcfbd0_0441_G08.ab1 |
| Ch5-1 | - | - | 17430237-17463778 | gcfbd0_1113B05 |
| Ch5-2 | SSR06660 | 10.9 | 21076611- 21112032 | gcfbd0_0472_A11.ab1 |
