# Supplementary figures and images for: A high-resolution cucumber cytogenetic map integrated with the genome assembly
Source: BMC Genomics. 2013 Jul 9;14:461. doi: 10.1186/1471-2164-14-461 (PMC3710503; doi:10.1186/1471-2164-14-461)

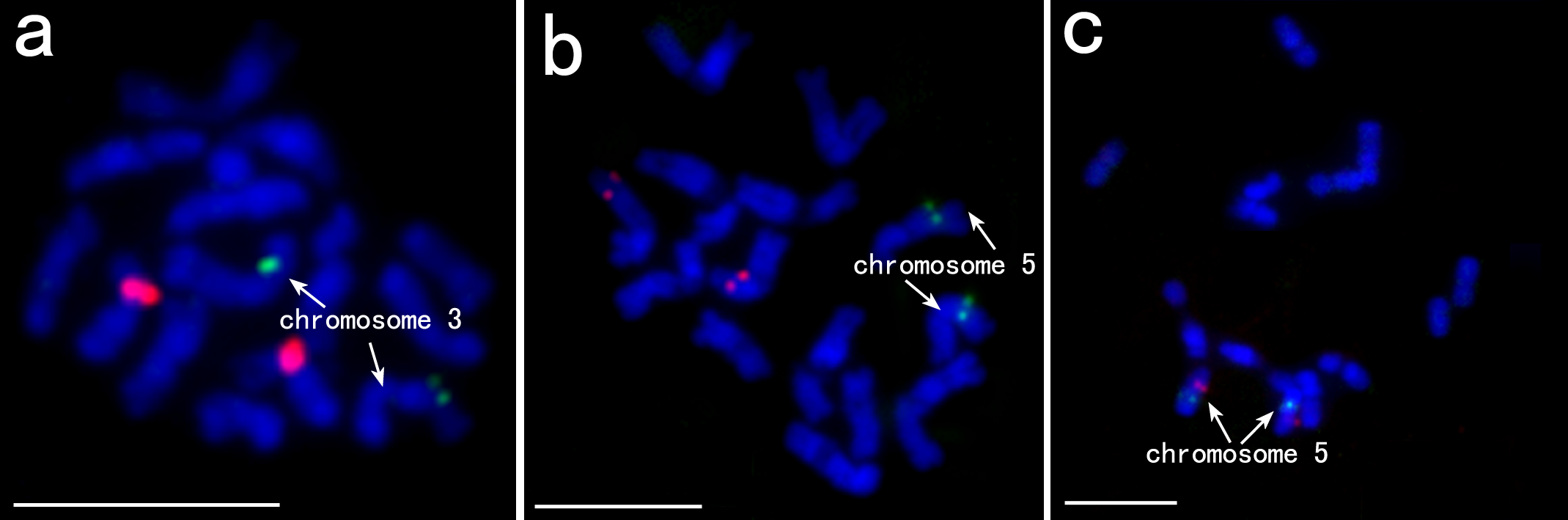

Supplement: Additional file 3 — FISH results of three identified misassembled clones. a The signals of Ch3-1 (red) and 3–2 (green) weren’t in the same chromosome pair. b The signals of Ch5-1 (red) and 5–10 (green) weren’t in the same chromosome pair. c Ch5-2 (red) was not in the long arm with 5–11 signals but in the short arm of chromosome 5. [file 1471-2164-14-461-S3.tiff]
