# Supplementary material for: A high-resolution cucumber cytogenetic map integrated with the genome assembly
Source: BMC Genomics. 2013 Jul 9;14:461. doi: 10.1186/1471-2164-14-461 (PMC3710503; doi:10.1186/1471-2164-14-461)
Supplement: Additional file 4 — Heterochromatin distribution on cucumber pachytene chromosomes 1, 3–5. [file 1471-2164-14-461-S4.doc]

| **Chr** | **Total heterochromatin ^a^ (%)** | **Telomeric heterochromatin ^a^ (%)** | **Centromeric and pericentromeric heterochromatin ^a^ (%)** |
| --- | --- | --- | --- |
|
| 1 | 14.6±0.5 | 4.5±0.2 | 10.1±0.3 |
| 3 | 7.5±0.5 | 3.8±0.2 | 3.7±0.2 |
| 4 | 18.8±0.9 | 4.4±0.3 | 14.4±0.6 |
| 5 | 8.3±0.1 | 4.6±0.2 | 3.7±0.3 |

^a^ (Heterochromatic region length/Total chromosome length) ×100
